# 

**Published:** 1886-04

**Authors:** 


### CONTENTS.

### COMMUNICATIONS.

Probable or Possible Poisoning from Dentist's Amalgam................. 161
Treatment of Pulpless Teeth..............................................168–171
Some Phenomena Observed in the Use of Antiseptics in Dental Surgery................................................................................ 175
Hydrogen Peroxide and its Utility........................................... 179
Death from Lodgment of a Gold Plate in the Bronchial Tubes ....... 184

### DISCUSSIONS.

Forty-Second Annual Meeting of the Mississippi Valley Association of Dental Surgeons.......... ................................................ 186

### PROCEEDINGS.

Minutes of the Mississippi Valley Association of Dental Surgeons .. 195
SALMAGUNDI........................................................ ........... 200

### CORRESPONDENCE.

The American Dental Association............................................ 206
Where shall the American Dental Association Meet in 1886?......... 209

### EDITORIAL.

Where to Meet.............................................................. 211
Michigan State Dental Society.................................. .............. 212
Illinois State Dental Society............................................... 213
Kansas State Dental Association............................................ 214
Obituary ........... ..................................................... ....... 214
Dr. E. F. Semple............................................................ 214

